# A High-Yield Co-Expression System for the Purification of an Intact Drs2p-Cdc50p Lipid Flippase Complex, Critically Dependent on and Stabilized by Phosphatidylinositol-4-Phosphate

**DOI:** 10.1371/journal.pone.0112176

**Published:** 2014-11-13

**Authors:** Hassina Azouaoui, Cédric Montigny, Miriam-Rose Ash, Frank Fijalkowski, Aurore Jacquot, Christina Grønberg, Rosa L. López-Marqués, Michael G. Palmgren, Manuel Garrigos, Marc le Maire, Paulette Decottignies, Pontus Gourdon, Poul Nissen, Philippe Champeil, Guillaume Lenoir

**Affiliations:** 1 Univ Paris-Sud, UMR 8221, Orsay, France; 2 CEA, iBiTec-S (Institut de Biologie et de Technologies de Saclay), SB^2^SM (Service de Bioénergétique, Biologie Structurale et Mécanismes), Laboratoire des Protéines Membranaires, Gif-sur-Yvette, France; 3 CNRS, UMR 8221, Gif-sur-Yvette, France; 4 Centre for Membrane Pumps in Cells and Disease – PUMPKIN, Danish National Research Foundation, Aarhus, Denmark; 5 Department of Molecular Biology and Genetics, Aarhus University, Aarhus, Denmark; 6 Department of Plant and Environmental Sciences, University of Copenhagen, Copenhagen, Denmark; 7 CNRS, Institut de Biochimie et Biophysique Moléculaire et Cellulaire, UMR 8619, Orsay, France; 8 Univ Paris-Sud, Orsay, France; College of Medicine, University of South Florida, United States of America

## Abstract

P-type ATPases from the P4 subfamily (P4-ATPases) are energy-dependent transporters, which are thought to establish lipid asymmetry in eukaryotic cell membranes. Together with their Cdc50 accessory subunits, P4-ATPases couple ATP hydrolysis to lipid transport from the exoplasmic to the cytoplasmic leaflet of plasma membranes, late Golgi membranes, and endosomes. To gain insights into the structure and function of these important membrane pumps, robust protocols for expression and purification are required. In this report, we present a procedure for high-yield co-expression of a yeast flippase, the Drs2p-Cdc50p complex. After recovery of yeast membranes expressing both proteins, efficient purification was achieved in a single step by affinity chromatography on streptavidin beads, yielding ∼1–2 mg purified Drs2p-Cdc50p complex per liter of culture. Importantly, the procedure enabled us to recover a fraction that mainly contained a 1∶1 complex, which was assessed by size-exclusion chromatography and mass spectrometry. The functional properties of the purified complex were examined, including the dependence of its catalytic cycle on specific lipids. The dephosphorylation rate was stimulated in the simultaneous presence of the transported substrate, phosphatidylserine (PS), and the regulatory lipid phosphatidylinositol-4-phosphate (PI4P), a phosphoinositide that plays critical roles in membrane trafficking events from the *trans*-Golgi network (TGN). Likewise, overall ATP hydrolysis by the complex was critically dependent on the simultaneous presence of PI4P and PS. We also identified a prominent role for PI4P in stabilization of the Drs2p-Cdc50p complex towards temperature- or C_12_E_8_-induced irreversible inactivation. These results indicate that the Drs2p-Cdc50p complex remains functional after affinity purification and that PI4P as a cofactor tightly controls its stability and catalytic activity. This work offers appealing perspectives for detailed structural and functional characterization of the Drs2p-Cdc50p lipid transport mechanism.

## Introduction

An essential feature of every eukaryotic cell is the asymmetric distribution of phospholipids between the two leaflets of membranes from the late secretory pathway. For instance phosphatidylserine (PS) is highly restricted to the inner leaflet of the plasma membrane [1]. Inside the cell, this specific distribution is important, because the negatively charged headgroup of PS is for instance the target of C2 domain-containing proteins involved in key cellular processes like protein phosphorylation or membrane fusion [2], [3], and more generally recruits various soluble proteins to the membrane via their polybasic motifs [4], [5], [6]. PS also affects the function of a number of transmembrane proteins [7]. Conversely, appearance of PS outside the cell is an early indicator of apoptosis [8] and a signal to initiate blood clotting [9].

PS distribution is therefore regulated tightly and energy-dependent transporters, the so-called flippases, have been postulated to transfer PS (as well as other phospholipid species) from the exoplasmic to the cytosolic leaflet of cell membranes. Recent data have revealed that flippases are members of the P4 subfamily of P-type ATPases (P4-ATPases) (*e.g.* [10] for review), a large family of ATP-driven membrane transporters (divided in 5 subfamilies, P1 to P5) that pump various substrates across cell membranes. All P-type ATPases share a common feature, namely the ability to form an acid-stable phosphorylated intermediate during their transport cycle. Although most P-type ATPases characterized to date have been shown to transport cations across membranes, P4-ATPases likely transport phospholipids, thereby maintaining phospholipid asymmetry in cell membranes. Studies in yeast revealed a critical role for P4-ATPases in various vesicular transport pathways [11]. For example, the yeast P4-ATPase Drs2p is required for budding of clathrin-coated vesicles at the TGN [12] and for bi-directional transport between early endosomes and the TGN [12], [13], [14]. To explain the membrane trafficking defects that occur after inactivation of P4-ATPases in yeast, several hypotheses have been raised: P4-ATPases might serve as a platform for the recruitment of proteins more directly involved in vesicular transport (*e.g.* Gea2p [15], [16]), or the P4-ATPase-catalyzed enrichment of specific lipids (such as PS) in the cytosolic leaflet might control the recruitment of proteins involved in vesicle formation, or the mere imbalance in phospholipid number caused by P4-ATPase-catalyzed lipid translocation would be directly involved in triggering initial membrane bending and subsequent vesicle formation – an early hypothesis supported by recent results from the Tanaka group [17], [18].

High-resolution crystal structures from most P-type ATPase subfamilies (i.e. P1, P2, and P3) have already been obtained (*e.g.* [19], [20], [21], [22]) and they indicate a common overall domain organization, with three cytoplasmic domains called the N-domain (nucleotide binding), the P-domain (phosphorylation), and the A-domain (actuator), as well as six to twelve α-helices comprising the membrane-embedded part of the enzyme [23]. Based on sequence alignments, the overall structure and the catalytic mechanism of P4-ATPases are expected to resemble those of P2-ATPases [24], and like some other P-type ATPases, P4-ATPases form heteromeric complexes with an additional protein subunit, in this case of the Cdc50 family. However, despite these similar features, the likely transport substrates of P4-ATPases (phospholipids) are very different from those of the classical cation-translocating ATPases, and the position of a phospholipid binding site as well as the nature of the transport pathway remain open questions [25], [26], [27], [28]. Hence, detailed structural and functional information is required to decipher the transport mechanism of P4-ATPases.

Quantitative purification in an active form is a prerequisite for detailed studies of any enzyme. For this purpose, the yeast Drs2p-Cdc50p lipid flippase complex has been overexpressed in yeast. Although previous studies described partial functional characterization of this purified flippase, only small amounts of Cdc50p were found to be attached to Drs2p [29], [30]. There has also been some ambiguity about whether for purification in an active form, the Drs2p protein should preferably be tagged at its N-terminus or at its C-terminus [30], [31], and in many instances, N-terminal and/or C-terminal extensions of P-type ATPases have indeed been suspected or found to have auto-inhibitory or regulatory roles [21], [32], [33]. Here, we shortly reexamine the effects of tagging the proteins at their N-terminal or C-terminal ends, and then describe an efficient single-step procedure for purifying a stoichiometric Drs2p-Cdc50p complex in significant amounts. Such purification enabled us to characterize some of the functional properties of the purified complex, namely the dependence on specific lipids of its phosphorylation and dephosphorylation properties, its overall ATP hydrolysis rate, and its stability.

## Materials and Methods

### Materials

Products for yeast and bacteria cultures were from Difco (BD Biosciences). The Biotin probe (Avidin coupled to HRP) was from Sigma-Aldrich, the Histidine probe (India Hisprobe-HRP) was from Thermo Fischer Scientific, and the rabbit polyclonal antibody directed against the large cytosplasmic insertion located between transmembrane helices 4 and 5 of Drs2p (corresponding to P- and N-domains) was kindly provided by Dr T. Graham [34]. Goat anti-rabbit secondary antibody coupled to HRP was from Bio-Rad. DDM (n-dodecyl-β-D-maltopyranoside) was from Affymetrix (Anatrace detergents and lipids, United Kingdom), and octaethylene glycol mono-n-dodecyl ether (C_12_E_8_) was from Nikkol Chemical (Tokyo, Japan). POPC (1-palmitoyl-2-oleoyl-*sn*-glycero-3-phosphocholine), DOPC (1,2-dioleoyl-*sn*-glycero-3-phosphocholine), POPE (1-palmitoyl-2-oleoyl-*sn*-glycero-3-phosphoethanolamine), POPS (1-palmitoyl-2-oleoyl-*sn*-glycero-3-phospho-L-serine), DMPS (1,2-dimyristoyl-*sn*-glycero-3-phospho-L-serine), and PI4P were from Avanti Polar Lipids (COGER, France). Streptavidin-Sepharose resin was from GE Healthcare. Protino Ni^2+^-TED silica resin was from Macherey-Nagel (France). YM100 ultrafiltration units were from Amicon systems (Millipore). TSK-3000SW silica gel column was purchased from Tosoh Biosciences (Tessenderlo, Belgium). Products for SDS-PAGE, haloalkanes-containing gels (TGX Stain-Free Precast 4-20% Gels), and gel filtration standards were from Bio-Rad (Marnes-la-Coquette, France). BSA (albumin fraction V) was from Roth Sochiel (France). [γ-^32^P]ATP was from PerkinElmer Life Sciences (catalog number BLU002A). Glass fiber A/E filters (1 µm porosity) were from Pall Corporation, and cellulose GS filters (0.22 µm porosity) were from Millipore. The EDTA-free SIGMAFAST protease inhibitors tablets were from Sigma-Aldrich (Saint-Quentin Fallavier, France). Most of the other chemical products were purchased from Sigma-Aldrich. Rabbit sarcoplasmic reticulum (SR) membranes were prepared as described previously [35].

### Yeast strains and plasmids

The *Saccharomyces cerevisiae* W303.1b/*GAL4* (*a, leu2-3, his3-11, trp1-1::TRP1-GAL10-GAL4, ura3-1, ade2-1, can^r^, cir^+^*) yeast strain was the same as previously described [36]. The *Δdrs2* and *Δcdc50* deletion mutants were created in W303.1b/*GAL4* background using a *loxP-HIS3-loxP* cassette, as described [37]. Plasmid pYeDP60 was generously given by Denis Pompon (LISBP, Toulouse, France). Plasmids allowing the expression of either Bad-tagged Drs2p or His_10_-tagged Cdc50p alone, as well as co-expression plasmids allowing coordinated overexpression of Bad-tagged Drs2p and His_10_-tagged Cdc50p, with the tags at either the N-terminus or the C-terminus (with a Tobacco Etch Virus (TEV) cleavage site between Drs2p and its tag, and with or without a TEV cleavage site between Cdc50p and its tag), were prepared as described previously [36], [38]. When present, the cleavage site was flanked by 2 glycines toward the tag and 4 glycines toward *DRS2* or *CDC50*. Site-directed mutagenesis was performed using the mega-primer method for D560N [39], or the QuickChange site-directed mutagenesis system for E342Q.

### Functional complementation of Δdrs2 and Δcdc50 growth defect

W303.1b/*GAL4* cells were transformed with the desired plasmid according to the lithium-acetate/single-stranded carrier DNA/PEG method [40]. Transformants were grown in a liquid glucose-containing medium, S6A (0.1% (w/v) bacto casamino acids, 0.7% (w/v) yeast nitrogen base, 2% (w/v) glucose, 20 µg/mL adenine), for 24 hours at 28°C, and then serially diluted (to 0.02, 0.001, or 0.0002 OD_600_) with a galactose-containing medium, S5A (0.1% (w/v) bacto casamino acids, 0.7% (w/v) yeast nitrogen base, 2% (w/v) galactose, 20 µg/mL adenine), supplemented with 1% (w/v) fructose to enable yeast growth at a fair rate. 5-µl drops were spotted onto S5A plates (S5A medium + 2% (w/v) agar) supplemented with 1% (w/v) fructose, and incubated at 20°C for 5–6 days or at 28°C for 2–3 days.

### Expression of Drs2p-Bad and Cdc50p-His_10_ in Fernbach flasks and yeast membrane preparation

Yeasts were transformed using the lithium-acetate/single-stranded carrier DNA/PEG method [40]. Yeast cultures, clone selection, recombinant protein expression and membrane preparation were performed as described previously [36], [38]. Briefly, yeast growth took place in a glucose-containing rich growth medium at 28°C for 36 h, whereas expression of the proteins of interest took place during an additional 18 h in the presence of galactose at 18°C. Yeast cells were harvested by centrifugation, washed with the appropriate buffer, and subsequently broken with glass beads using a “Pulverisette 6” planetary mill (Fritsch). The crude extract was then spun down at 1,000 g for 20 min at 4°C, to remove cell debris and nuclei. The resulting supernatant was centrifuged at 20,000 g for 20 min at 4°C, yielding S2 supernatant and P2 pellet. The S2 supernatant was further centrifuged at 125,000 g for 1 h at 4°C. The resulting P3 pellet was finally resuspended at about 30 mg/mL of total protein in HEPES-sucrose buffer (20 mM HEPES-Tris pH 7.4, 0.3 M sucrose, 0.1 mM CaCl_2_). We estimated, by western-blotting, that the proteins in the P3 fraction comprise about 3% of Drs2p [38].

### Protein estimation and detection

In the membrane fractions, protein concentrations were measured with the bicinchoninic acid procedure [41] in the presence of 2% (w/v) SDS, using bovine serum albumin as a standard. For the purified sample, its UV absorption was measured at 280 nm, and converted into mg protein/mL on the basis of the optical extinction coefficient predicted from the sequences (www.expasy.org) for an equimolar mixture of Drs2p and Cdc50p. According to this prediction, 1 mg/mL complex should result in an OD of about 1.3 at 280 nm (for a 1-cm optical path). Alternatively, purified Drs2p was compared with known concentrations of Ca^2+^-ATPase from sarcoplasmic reticulum membranes (SERCA1a) using Coomassie Blue stained gels. Protein concentrations estimated by the two methods were similar.

For electrophoretic separation and identification, proteins were loaded onto SDS-PAGE [42]. Proteins on the gels were then either stained with Coomassie Blue (or silver nitrate), or transferred to PVDF membranes. Detection of the biotinylated Drs2p-Bad was performed using a Biotin probe (at a 1/25,000 dilution), detection of total Drs2p was performed using an α-Drs2p antibody (at a 1/5,000 dilution), and detection of Cdc50p-His_10_ was performed using a Histidine probe (at a 1/2,000 dilution).

In a few cases, before their staining with Coomassie Blue, proteins were visualized in-gel by light-induced specific chemical modification of their Trp residues by a haloalkane, which makes these modified Trp residues fluoresce at a convenient visible wavelength [43], [44].

Streptavidin resin samples were heated up to 90°C before loading onto SDS-PAGE (this was not the case for other samples) in order to allow dissociation of the strong SDS-resistant interaction of avidin with the biotin bound to the Bad-tagged Drs2p. This heating step is responsible for excessive formation of Drs2p aggregates in the corresponding samples (data not shown).

### Solubilization of P3 membranes and purification of the Drs2p-Cdc50p complex

P3 membranes obtained after co-expression of Drs2p and Cdc50p were diluted to 2 mg of total protein/mL (typically 120 mL) in ice-cold SSR buffer (Streptavidin Sepharose Resin buffer, containing 50 mM Mops-Tris at pH 7, 100 mM KCl, 20% (w/v) glycerol and 5 mM MgCl_2_), supplemented with 1 mM PMSF and an EDTA-free protease inhibitor mixture. DDM was added at a final concentration of 2 mg/mL (*i.e.* at a DDM: protein ratio of 1∶1, w:w) and the suspension was stirred gently on a wheel for 15 minutes at 4°C. Insoluble material was pelleted by centrifugation at 100,000 g for 1 h at 4°C. The supernatant, containing solubilized proteins, was applied onto a streptavidin-Sepharose resin (typically 2.4 mL of previously washed resin per 120 mL of solubilized material) and incubated for 2 hours at 4°C to allow binding of the Bad-tagged Drs2p to the resin. To eliminate unbound material, the resin was washed four times with three resin volumes of SSR buffer supplemented with 0.5 mg/mL DDM and 0.025 mg/mL phosphatidylserine (POPS), in the presence of 1 mM PMSF but in the absence of the SIGMA-FAST protease inhibitor cocktail. Subsequent cleavage by TEV (at 0.03 mg/mL of total volume, resin + buffer) of the cleavage site inserted between Drs2p and its Bad moiety allowed release of Drs2p from the resin and simultaneous removal of the deca-histidine tag on Cdc50p, by overnight incubation at 6°C.

For removal of the His_6_-tagged TEV protease, the streptavidin-purified fraction was mixed with dry Ni^2+^-TED resin in a 500∶1 Ni^2+^-TED:TEV ratio (w:w), and incubated for 45 minutes at 4°C with gentle mixing on a wheel. A TEV-free fraction was recovered by centrifugation at 500 g for 5 minutes.

For Size-Exclusion Chromatography (SEC)-HPLC, the streptavidin-purified sample was first incubated with Ni^2+^-TED, to remove the TEV protease, and then concentrated on YM100 ultrafiltration units (100 kDa cutoff). The YM100-concentrated fraction (about 4 mg/mL) was briefly centrifuged (120,000 g for 10 minutes in a Beckman Coulter TLA 100.3 rotor) to get rid of large aggregates. Aliquots (300 µL each) of this concentrated supernatant were applied onto a TSK-3000SW column. Chromatography was performed at a flow rate of 1 mL/min, with the SEC-HPLC mobile phase buffer corresponding to SSR buffer without glycerol, supplemented with 0.5 mg/mL DDM and 0.025 mg/mL POPS. In order to limit unspecific adsorption of the proteins, filters and columns had been rinsed with the detergent- and lipid- containing buffer. The SEC purified fractions were frozen in liquid nitrogen and stored at −80°C.

Standard soluble proteins (250 µL at 1 mg/mL) were used to calibrate the column, and DDM-solubilized SERCA1a was used as a control. For the latter purpose, SR membranes were diluted to 4 mg/mL in the mobile phase buffer supplemented with 1 mM Ca^2+^ and 40 mg/mL DDM. After 10 minutes incubation at 20°C, 550 µL of this suspension was centrifuged at 120,000 g for 10 minutes in a TLA 100.3 rotor, and 500 µL of the supernatant (solubilized material) was injected on the column.

### Analysis by Matrix-Assisted Laser Desorption/Ionization – Time of Flight (MALDI-TOF) mass spectrometry of the purified Drs2p-Cdc50p complex

Mass spectrometry analysis was first performed using a procedure previously developed for detergent-solubilized and SEC-purified intact membrane proteins [45], [46]. One microliter of the SEC purified sample was mixed with 3 µL of a saturated solution of sinapinic acid in 30% acetonitrile and 0.3% trifluoroacetic acid. One microliter of the mixture was loaded into a MALDI-TOF spectrometer (Perseptive Biosystems, Voyager DE-STR) equipped with a 337-nm nitrogen laser. Spectra were obtained in linear mode using delayed extraction. Mass range was from 30,000–200,000 Da (accelerating voltage of 25 kV). The external standards used for calibration were: aldolase (39,211 Da) and albumin (66,429 Da) (Proteomass MALDI-MS standards, Sigma). Mass spectrometry was also used for unambiguous identification of Drs2p and Cdc50p in the final purified sample, as follows. SEC-purified samples were first loaded onto a 7% SDS-PAGE, and the gel was stained with Coomassie Blue. Bands of interest were excised manually and destained with a 50∶50 mixture of acetonitrile:0.1 M NH_4_HCO_3_, and then submitted to tryptic digestion overnight at 37°C in 50 mM NH_4_HCO_3_. The supernatants were removed and peptides were extracted with 1% trifluoroacetic acid (TFA) then with 60% acetonitrile containing 1% (v/v) TFA. The combined extracts were evaporated to 2 µL, mixed with the same volume of half-saturated solution of α-cyano-4-hydroxycinnamic acid in 50% acetonitrile, 0.3% TFA, and submitted to MALDI-TOF mass spectrometry (Voyager DE-STR). Spectra were acquired in positive-ion reflector mode with delayed extraction, using six peptides of known masses as close external calibration standards. Proteins were identified using the Peptide Mass Fingerprint search program Mascot (http://www.matrixscience.com/search_form_select.html) reducing the NCBInr (release 20131113, 24201960 entries) to the *Saccharomyces cerevisiae* species. Two missed cleavage sites and possible oxidation of methionines were considered in searches.

### Phosphorylation from [γ-^32^P]ATP and turnover-dependent dephosphorylation

Transient formation of the phosphoenzyme intermediate in the Drs2p catalytic cycle was measured after incubation with [γ-^32^P]ATP followed by acid quenching, using a filtration protocol (see *e.g.* [47]).

**Figure 1:**
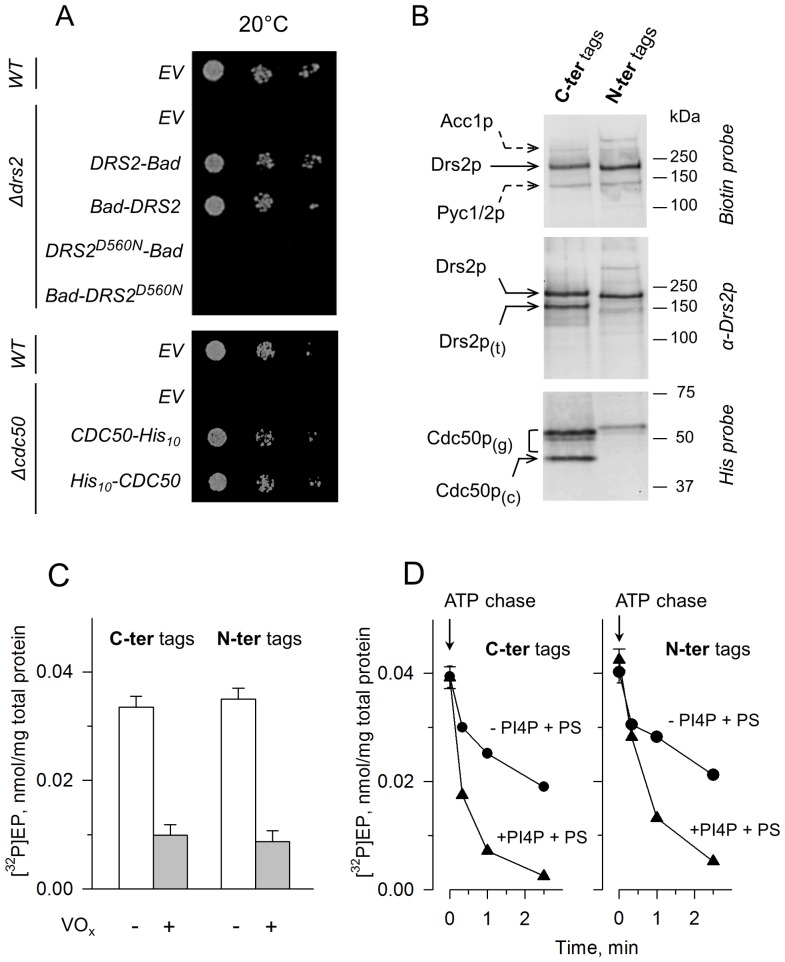
Co-expression in yeast and functional properties of C- or N-terminally tagged Drs2p and Cdc50p. **(A)** Functional complementation of the temperature-sensitive phenotype of *Δdrs2* and *Δcdc50* yeast cells. Yeast cells, either wild-type, *Δdrs2*, or *Δcdc50* mutants, were transformed with plasmids bearing either *DRS2* (WT or D560N) tagged with a sequence coding for Bad, or *CDC50* tagged with ten histidines (His_10_). Tags were inserted at the proteins C- or N-termini. Cells transformed with an empty vector (EV) were used as negative control. Serial dilutions of yeast cells were spotted on plates and incubated at the restrictive temperature of 20°C. **(B)** Co-expression of C-terminally or N-terminally tagged Drs2p and Cdc50p in P3 membranes, as analyzed by western-blotting. For detection using a Biotin or a Histidine probe (top and bottom gels, respectively), 1.5 µg of total protein was loaded per lane and 1 µg total protein was used for detection with an α-Drs2p antibody (intermediate gel). The biotin probe also weakly detected endogenous yeast proteins known to be biotinylated, Acc1p and Pyc1/2p (dashed arrows). Drs2p_(t)_, truncated form of Drs2p; Cdc50p_(g)_, glycosylated forms of Cdc50p [36]; Cdc50p_(c)_, core (unglycosylated) Cdc50p. **(C)** Phosphorylation from [γ-^32^P]ATP of P3 membrane fractions co-expressing the tagged Drs2p and Cdc50p proteins. Formation of the phosphoenzyme intermediate was measured in the absence or presence of 1 mM vanadate (open bars and grey bars, respectively). **(D)** Turnover-dependent dephosphorylation in the presence of DDM and POPS and in the presence (triangles) or absence (circles) of PI4P (see “Materials and Methods”).

For experiments with P3 membranes (Figure 1C and 1D), 40-µl samples at 0.5 mg/mL of total protein were pre-incubated on ice in buffer A (100 mM KCl, 5 mM Mg^2+^ and 50 mM Mops-Tris at pH 7), supplemented with 5 mg/mL DDM in the presence or absence of 0.25 mg/mL POPS or 0.25 mg/mL PI4P. In some cases, 1 mM orthovanadate was added. Phosphorylation was triggered by addition of 0.5 µM [γ-^32^P]ATP (at 0.25–1 mCi/µmol) on ice (to avoid excessive ATP hydrolysis by Drs2p-unrelated proteins in crude membranes), followed after 25 seconds by acid quenching (typically 1 mL of 500 mM trichloroacetic acid (TCA) + 30 mM H_3_PO_4_). Samples were left on ice for more than half an hour after quenching, a period sufficient for aggregation of the precipitated protein and therefore its retention by the filter (this aggregation period was critical in the presence of detergent). This was followed by filtration on either a glass fiber A/E filter or a nitrocellulose GSWP filter, and careful rinsing with dilute acid (50 mM TCA + 3 mM H_3_PO_4_). The kinetics of turnover-dependent dephosphorylation were measured by first phosphorylating the sample for 25 seconds on ice under the above conditions and then chasing ^32^P from the phosphoenzyme by transferring it into a tube pre-equilibrated at 37°C and containing concentrated non-radioactive Mg-ATP (so that its final concentration was 1 mM) for dephosphorylation during the desired time period.

As purification of the Drs2p-Cdc50p complex allowed us to get rid of most of the other ATP-hydrolyzing proteins present in crude membranes, in this case, we could improve our phosphorylation protocol compared with phosphorylation of Drs2p in membranes: instead of allowing phosphorylation to proceed on ice and only then transferring the sample at 37°C for dephosphorylation, we could perform both phosphorylation and dephosphorylation at the same temperature (30°C). The purified sample (typically 40 µL at 50–100 µg protein/mL), on ice, generally in SSR buffer in the presence of detergent and exogenous lipids, was supplemented with [γ-^32^P]ATP and immediately transferred to 30°C for phosphorylation, before classical acid quenching. Turnover-dependent dephosphorylation was measured by first phosphorylating the sample up to steady-state under the above conditions (typically 1 minute and a half) and then chasing ^32^P from the phosphoenzyme by simply adding concentrated non-radioactive Mg-ATP (so that its final concentration was 1 mM) for dephosphorylation during the desired time period at the same temperature (30°C). In some cases, phosphorylation and dephosphorylation were measured in a KNG buffer (SSR buffer containing 66 mM NaCl and 33 mM KCl instead of 100 mM KCl), with no obvious difference.

### ATPase activity of purified samples

P_i_ released from ATP as a result of Mg-ATP hydrolysis was deduced from colorimetric measurements, as described in [48]. In some cases, the purified Drs2p-Cdc50p complex was first pre-incubated with VO_x_, BeF_3_^−^, or AlF_4_^−^. Typically, the enzyme (at about 50 µg Drs2p/mL) was supplemented with Mg-ATP (generally 1 mM) and incubated at 30°C for various periods. At the desired time point, 33 µL aliquots were withdrawn and quenched with 17 µL of 10% SDS (plus antifoam). P_i_ in these samples was subsequently revealed by adding ammonium-molybdate and zinc acetate at pH 5.0 [49], loading 96-well plates with these samples and measuring the resulting blue color at 850 nm, after 1–2 hours at room temperature. In some cases, ATPase activity was measured at lower concentrations of ATP, with 0.1 mg/mL pyruvate kinase and 1 mM phosphoenolpyruvate as a regenerating system. ATPase activities were measured in KNG buffer or in SSR buffer, with no difference.

Throughout this paper, the average of two to three independent data points (± SD) is displayed.

## Results

### Co-expression in yeast and functional properties of C- or N-terminally tagged Drs2p-Cdc50p complexes

In our previous work, we had chosen to introduce a Bad (Biotin Acceptor Domain) tag and a His_10_ tag at the C-termini of Drs2p and Cdc50p, respectively, for their overexpression in yeast [36]. However, the position of affinity tags on Drs2p has been suggested to affect the activity of the resulting purified sample [30], [31]. In the present work, we therefore investigated in more detail the result of an N-terminal location for these tags. We first looked at the ability of the two types of constructs to restore normal growth phenotypes in yeast strains deficient in one or the other of these proteins. When *Δcdc50* cold-sensitive yeast strains were transformed with an expression plasmid bearing the sole *CDC50* gene tagged at either its 5′ or 3′ end, growth at 20°C was restored in both cases (Figure 1A, bottom). When *Δdrs2* cold-sensitive yeast strains were transformed with a plasmid bearing the sole *DRS2* gene, tagged at either its 5′ or 3′ end, growth at 20°C was also restored in both cases (Figure 1A, top), in agreement with a previous report in which a different set of tags was used [30]. However, expression of an inactive variant, bearing a mutation at the catalytic aspartate (D560N), did not restore growth at 20°C. Thus, at this step, the location of the tag did not appear to be critical, and we attempted to overexpress both types of constructs using a co-expression strategy where Drs2p and Cdc50p are both coded on the same plasmid and where protein expression may be achieved at high yeast cell densities [36].

After growth and expression, membranes were separated by differential centrifugation, and fractions recovered after high-speed centrifugation (P3) were submitted to SDS-PAGE. Use of a Biotin probe revealed similar expression levels for the Bad-tagged Drs2p, irrespective of the tag location. Endogenous proteins, *e.g.* Acc1p and Pyc1/2p, known to be biotinylated in yeast, were also detected (Figure 1B, top). For the construct with C-terminal tags, use of an antibody directed against Drs2p (kindly provided by Dr Graham) revealed two bands for Drs2p (Figure 1B, middle). This indicated the presence of a truncated form of Drs2p (Drs2p_(t)_), lacking its C-terminally tagged end (since this degraded form was not detected by the Biotin probe). For this construct, use of a His probe also indicated that a significant fraction of C-terminally tagged Cdc50p was not glycosylated (Figure 1B, bottom). In contrast, for the construct with N-terminal tags, truncation of Drs2p was significantly less prominent and the relative contribution of the non-glycosylated Cdc50p was also reduced (Figure 1B, middle and bottom). The fact that the truncated form of Drs2p was not revealed by the Biotin probe using either N- or C-terminally tagged constructs suggests that it results from degradation at both its N- and C-termini.

Incidentally, note that having the Bad tag at the C-terminus of Drs2p makes migration of the full-length Drs2p slightly slower than when this tag is attached at the N-terminus (Figure 1B, top and middle). Since subsequent Bad tag removal by TEV protease results in virtually identical mobilities (data not shown), this implies that the mere location of the tag affects the residual partial folding of Drs2p in SDS. The fact that SDS only partially unfolds proteins has already been documented for many other proteins [50]. The same fact is observed for Cdc50p tagged with His_10_, although in the opposite direction (Figure 1B, bottom). As subsequent tag removal again results in identical electrophoretic mobilities for both constructs (data not shown), this cannot be explained by different glycosylation levels.

We then compared the functional properties of C- or N-terminally tagged proteins. Two of these properties can be tested with crude P3 membranes, namely the ability of Drs2p to become phosphorylated and the rate of its dephosphorylation. In spite of their different susceptibility to proteolysis, C-terminally and N-terminally tagged Drs2p had a similar vanadate-sensitive phosphoenzyme level at steady-state (on a nmol ^32^P per mg total protein basis) (Figure 1C). Moreover, the ability of phosphatidylinositol-4-phosphate (PI4P, a phosphoinositide previously shown to be closely associated with the flippase activity of Drs2p [16], [31], [36]) to stimulate dephosphorylation of Drs2p in the presence of phosphatidylserine was also observed in both cases (Figure 1D).

In the rest of this study, we focused on Drs2p-Cdc50p proteins overexpressed using the N-terminally tagged constructs, because of the lower extent of undesired proteolysis and of the more complete glycosylation in this case.

### Streptavidin-based single-step purification of Drs2p-Cdc50p complex

**Figure 2:**
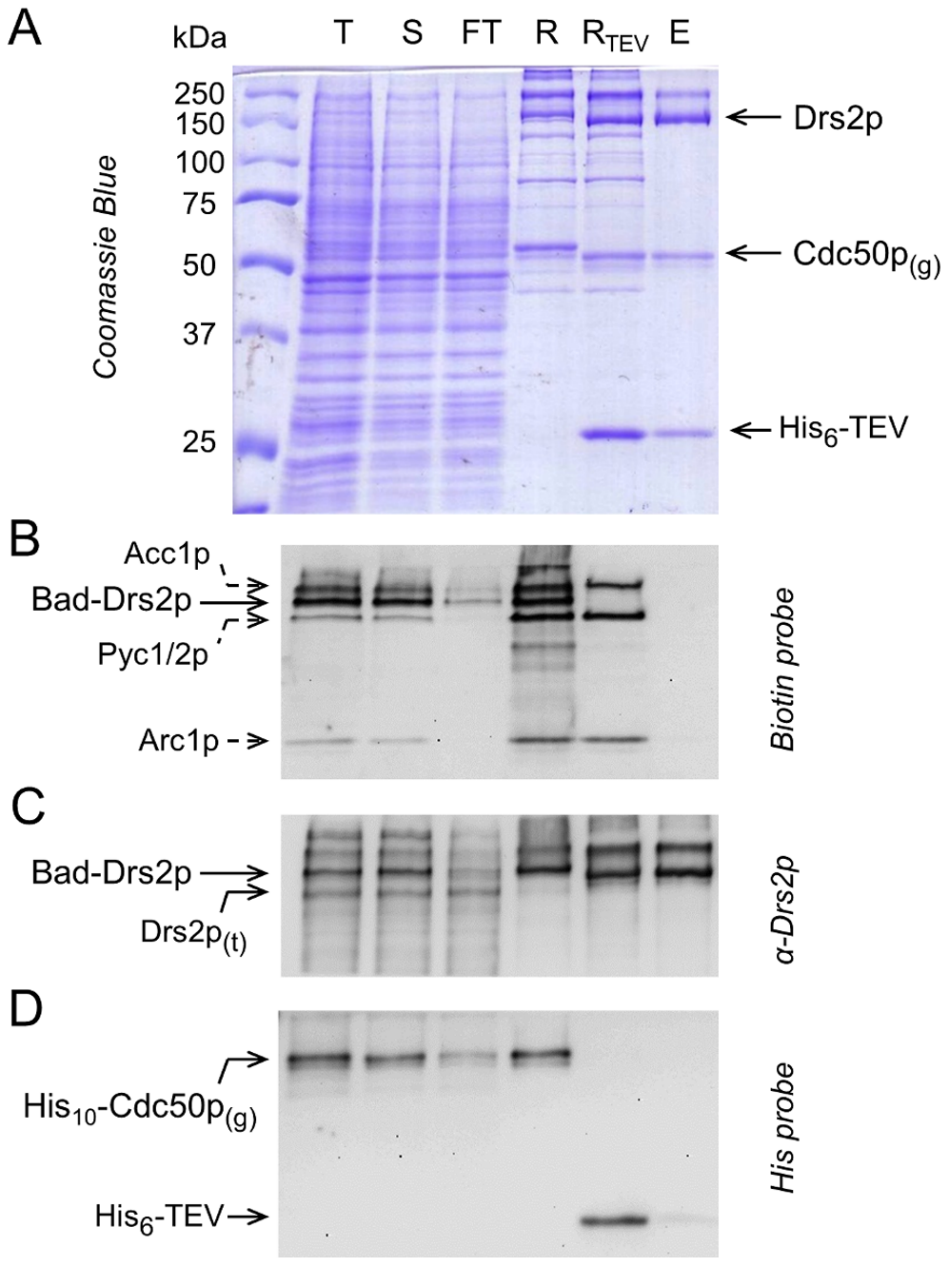
Streptavidin-based purification of Drs2p and Cdc50p. N-terminally Bad-tagged Drs2p and His_10_-tagged Cdc50p were purified onto a streptavidin column, and eluted by TEV cleavage. **(A)** Coomassie Blue stained 10% SDS-PAGE. **(B, C, D)** Immunodetection using a Biotin probe, a α-Drs2p antibody and a Histidine probe, as indicated. T, total membranes before solubilization; S, DDM-solubilized fraction; FT, flow-through; R, Streptavidin Sepharose resin before addition of TEV; R_TEV_, Streptavidin Sepharose resin after incubation with TEV for 16 h at 6°C; E, fraction eluted from the resin after incubation with TEV. Drs2p_(t)_, truncated form of Drs2p; Cdc50p_(g)_, glycosylated form of Cdc50p; His_6_-TEV, N-terminally hexahistidine-tagged TEV.

In previous experiments performed with solubilized crude membranes, we found that for protection of Drs2p from detergent-induced irreversible inactivation, DDM at a moderate concentration was one of the less deleterious detergents. DDM, for instance, was superior to C_12_E_8_ or Triton X-100 [36], and our purification procedure was therefore developed in the presence of DDM. Purification of the Drs2p-Cdc50p complex was achieved thanks to the strong interaction between the biotinylated Bad tag of Drs2p and an avidin-based resin. Figure 2 shows typical data obtained from N-terminally tagged constructs.

Solubilization of the Bad-tagged Drs2p and the His_10_-tagged Cdc50p was almost complete (compare T and S lanes in Figure 2B–2D). Most of the solubilized full-length Bad-Drs2p bound to the streptavidin resin (compare S and FT lanes in Figure 2B and 2C). In contrast, most of the solubilized truncated Drs2p (Drs2p_(t)_, Figure 2C) remained in the streptavidin flow-through, in agreement with the above suggestion that the truncated form of Drs2p has lost some of both its N- and C-termini.

The resin only retained a limited number of proteins (R lane in Figure 2A), primarily the full-length Bad-tagged Drs2p as well as endogenous, biotinylated yeast proteins (R lane in Figure 2B and 2C). Remarkably, the resin also retained most of His_10_-tagged Cdc50p (S, FT, and R lanes in Figure 2D), demonstrating that thanks to our co-expression strategy the N-terminally tagged Cdc50p is not produced in large excess compared with Drs2p, and strongly suggesting, in addition, that the majority of the expressed Cdc50p still interacts with Drs2p in the solubilized membranes.

Incubation of the resin with TEV protease (targeting a TEV protease cleavage site inserted between Drs2p and its Bad tag) made it possible to release Drs2p from the streptavidin beads. Cleavage was essentially complete for Bad-tagged Drs2p (Figure 2B). This was accompanied by a slight increase in the electrophoretic mobility of Drs2p (Figure 2A and 2C), while the addition of TEV did not affect the endogenous yeast proteins also retained by the resin. Cleavage was also complete for Cdc50p (Figure 2D), again with a distinct change in its migration rate (Figure 2A).

According to SDS-PAGE, the fraction eluted from the streptavidin beads contained highly pure and tag-free Drs2p and Cdc50p proteins, as well as some TEV protease (E lane in Figure 2). Note that TEV remained bound to the streptavidin resin to a significant extent (compare lanes R_TEV_ and E in Figure 2A and 2D), presumably due to binding of the protease, after its proteolytic action, to the six residues that follow the resin-bound N-terminal Bad tag (consistent with this, the amount of TEV found in the eluate was larger when C-terminal constructs were used, data not shown). In the final purified sample, the concentration of Drs2p was about 0.2–0.3 mg/mL, yielding a total of 1–2 mg purified Drs2p-Cdc50p for a 1-L yeast culture.

**Figure 3:**
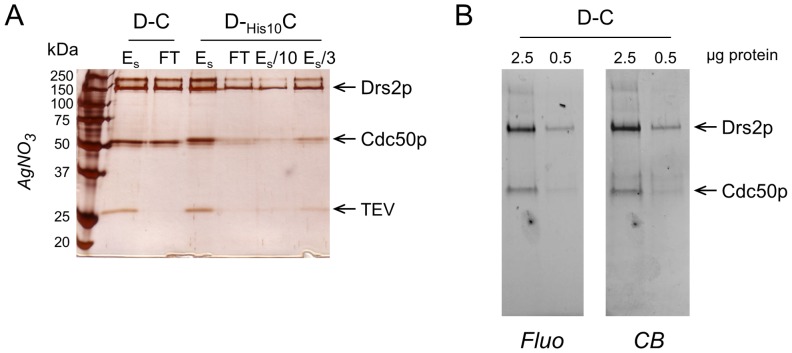
The vast majority of Drs2p is in complex with Cdc50p, in a 1/1 stoichiometry. **(A)** Yeasts were transformed either with a regular Bad-Drs2p/His_10_-Cdc50p construct (with N-terminal tags) or with a related construct in which the TEV cleavage site between the His_10_ tag and Cdc50p had been omitted. This resulted, after streptavidin-based purification, in either the classical sample (D–C) or in a sample where Cdc50p remains tagged with His_10_ (D-_His10_C). For both samples, the streptavidin-eluted fractions were diluted 5-fold (to about 70 µg/mL) in KNG buffer supplemented with 1 mg/mL DDM, 0.025 mg/mL PS and 0.025 mg/mL PI4P, and 300 µL of each diluted sample was mixed with 5 mg of dry Ni^2+^-TED resin (previously washed with the dilution buffer) and incubated on a wheel for 45 minutes in the cold room. Initial diluted samples (E_s_), and unbound material (FT), were loaded onto a 10% SDS-PAGE and stained with silver nitrate. For D-His_10_C, the E_s_ sample was further diluted 3-fold and 10-fold and aliquots (E_s_/10 and E_s_/3) were loaded for comparison with the FT sample. **(B)** 2.5 µg and 0.5 µg of streptavidin-purified Drs2p-Cdc50p complex were loaded onto a haloalkane-containing 4–20% gradient gel for both in-gel fluorescence analysis (left, Fluo) and subsequent Coomassie Blue staining of the same gel (right, CB).

Final removal of the His_6_-tagged TEV protease was easily done by incubating the streptavidin-purified eluate (E_s_) with a nickel affinity resin, with only little non-specific loss of Drs2p and Cdc50p (Figure 3A).

The fact that some Cdc50p is found in the fraction eluted from streptavidin beads does not necessarily imply that all Drs2p molecules are in complex with Cdc50p (see for instance [29] where after a related purification attempt only 10% of purified Drs2p was found to interact with Cdc50p). To determine how much of the purified Drs2p is bound to its partner subunit, we co-expressed, together with Drs2p, a version of Cdc50p lacking the TEV-cleavage site between the His_10_ tag and Cdc50p. In that case, purification on the streptavidin resin followed by TEV protease action results in a Drs2p-_His10_Cdc50p sample (D-_His10_C), instead of the previous tag-free Drs2p-Cdc50p sample (D–C). Subjecting this D-_His10_C sample to the Ni^2+^-TED step should allow the Ni^2+^-TED resin to capture Drs2p molecules that are in complex with His_10_-Cdc50p, while leaving Cdc50p-unbound Drs2p molecules in the flow-through, and therefore should make it possible to quantify the fraction of Drs2p molecules that are not associated with Cdc50p.

In such an experiment, only a small proportion of the His_10_-tagged Cdc50p eluted from the streptavidin resin was recovered in the Ni^2+^-TED flow-through (Figure 3A). Simultaneously, only a small proportion of Drs2p was present in this flow-through, indicating that the vast majority of Drs2p molecules are in tight complex with Cdc50p. Estimation of the proportions of His_10_-Cdc50p and Drs2p remaining in the Ni^2+^-TED flow-through was performed by comparison with various dilutions of the initial streptavidin eluate E_s_ (Figure 3A, lanes E_s_/10 and E_s_/3), thus avoiding possible errors due to non-linearity of the silver staining procedure. It appears that the amount of Drs2p and Cdc50p in the flow-through corresponds for both proteins to about 20% of the initial total amount. This estimation was supported by western-blot analysis of the same samples (Figure S1). Therefore, at least 80% of Drs2p is bound to Cdc50p in the “E_s_” purified fraction. Note that in this experiment, the streptavidin eluate had been diluted five-fold before interaction with Ni^2+^-TED. It is therefore expected that the fraction of Cdc50p-bound Drs2p will be even greater than 80% in the original purified fraction, as dilution might lead to dissociation of the Drs2p-Cdc50p heterodimer.

In our purified material, there is therefore neither excess Cdc50p (because of the Bad-dependent purification procedure), nor excess Cdc50p-unbound Drs2p (Figure 3). The stoichiometry of the complex itself was estimated using in-gel fluorescence after modification of the protein Trp residues by a haloalkane [43], [44]. Indeed, assuming that all protein Trp residues behave similarly in the denaturing SDS-PAGE environment, the fluorescence intensities associated with Drs2p and Cdc50p bands should reflect their abundance. In-gel fluorescence quantification (Figure 3B, left) indicated a 3-fold higher fluorescence intensity for Drs2p than for Cdc50p, as expected for a 1∶1 complex (mol:mol) on the basis of the molecular weights and respective Trp contents of Drs2p (154 kDa and 18 Trp) and Cdc50p (45 kDa for the non-glycosylated protein and 6 Trp). Note that estimating this stoichiometry on the basis of quantification of the Coomassie Blue stained corresponding bands (Figure 3B, right) leads to a similar 1∶1 Drs2p:Cdc50p stoichiometry (mol:mol) in the streptavidin eluate.

### Further analysis by size-exclusion chromatography (SEC) and mass spectrometry

**Figure 4:**
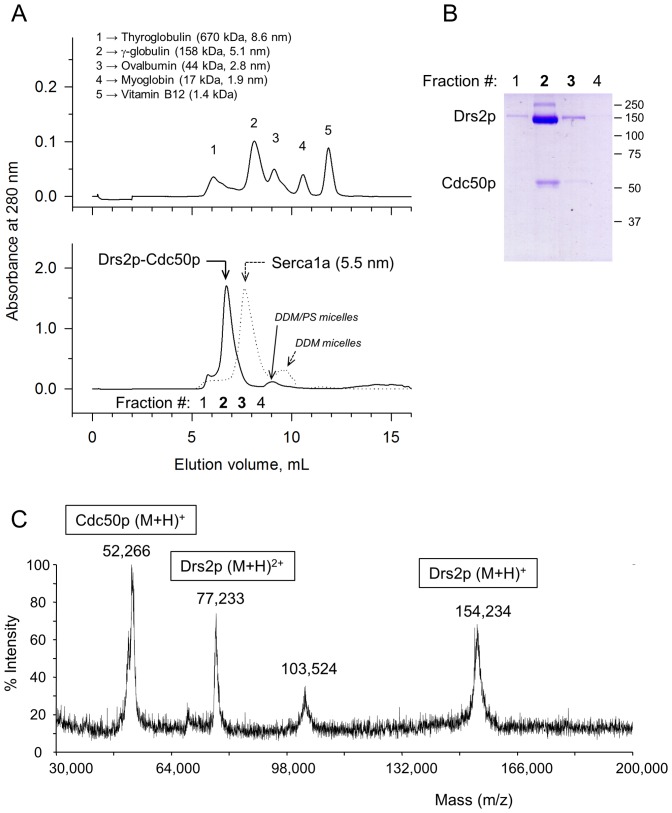
Size-exclusion chromatography and mass spectrometry analysis of the purified Drs2p-Cdc50p complex. **(A)** Top: calibration of the TSK-3000SW silica gel column with gel filtration standards. The elution volume of Thyroglobulin corresponds to the dead volume (V_0_), while Vitamin B12 is eluted at a volume close to the total volume (V_t_). Bottom: size-exclusion chromatography profile of the streptavidin-purified Drs2p-Cdc50p complex (continuous line). The streptavidin-purified and Ni^2+^-TED-treated fraction was first concentrated on YM100 filters and 300 µL was then injected on the column. Fractions 1–4 were collected. The dotted line shows the behavior of a control DDM-solubilized SR SERCA1a sample (500 µL at 4 mg/mL was injected). **(B)** Analysis of the collected fractions on an 8% Coomassie Blue stained SDS-PAGE. **(C)** An aliquot of the SEC-eluted sample was submitted to mass spectrometry analysis. Spectra were acquired on a range of m/z values from 30,000 to 200,000.

After concentration on a 100 kDa centrifugal filter device, the purified Drs2p-Cdc50p sample was analyzed by SEC on a TSK-3000SW silica gel column, in the presence of DDM. Judging from the elution positions of soluble standards (Figure 4A, top) or of DDM-solubilized SERCA1a (110 kDa, 5.5 nm Stokes radius, Figure 4A, bottom), the main elution peak (Figure 4A, bottom) was found in the expected region. Its fairly symmetrical shape indicated a good homogeneity and the initial sample apparently contained only a very limited amount of aggregated material eluting in the column void volume. Cdc50p eluted together with Drs2p, as judged from SDS-PAGE analysis of eluted fractions, confirming the tight association of the two protein subunits (Figure 4B).

We also submitted the SEC-purified sample to complementary analysis by MALDI-TOF mass spectrometry, without any preliminary trypsinolysis. As displayed in Figure 4C, only a few peaks showed up, and they were in the expected range of molecular masses. One, presumably corresponding to the singly charged form of Drs2p, had an m/z at 154,324 Da, and another one, presumably corresponding to the same peptide with two positive charges, had an m/z at 77,233 (77,233×2  =  154,466). 154,324 and 154,466 are close to the theoretical mass expected for recombinant Drs2p, namely 154,050 Da (including what is left from the N-terminally located TEV cleavage site (one glycine), plus four glycines used as a linker between the TEV cleavage site and the N-terminus of Drs2p). A major peak also showed up with an m/z at 52,266, presumably corresponding to the monoionized form of glycosylated Cdc50p (since the molecular mass of recombinant non-glycosylated Cdc50p is only 45,136, again including the linker glycines). The species detected at m/z of ∼103,524 presumably corresponds to dimers (52,266×2  =  104,532) of this glycosylated Cdc50p. The SEC-purified sample was also treated with trypsin, which allowed identification of several peptides corresponding to Drs2p and Cdc50p (data not shown).

The purification procedure described here allows us to recover a sample which contains Drs2p and Cdc50p in complex, most likely in a 1∶1 stoichiometry. Starting from 240 mL of yeast P3 membranes at 2 mg protein/mL (prepared from 2.4 L of yeast culture), we recovered 4 mL of a SEC-purified fraction containing about 0.6 mg.mL^−1^ of protein (as estimated from its absorbance at 280 nm), i.e. about 2.4 mg of purified Drs2p-Cdc50p (out of which ∼1.8 mg is Drs2p). We could therefore recover about 1 mg of SEC-purified complex per liter of yeast culture, with an estimated purification yield of 13% assuming 3% Bad-Drs2p enrichment in the initial P3 fraction (see [36]).

### The purified Drs2p-Cdc50p complex retains a sub-micromolar apparent affinity for phosphorylation from ATP, as well as PI4P-stimulated dephosphorylation

At this step, we turned to functional characterization, and first asked whether the purified Drs2p-Cdc50p complex has retained the phosphorylation and dephosphorylation properties previously documented in crude membranes (Figure 1D and [36]). These analyses were generally performed with the streptavidin-purified Drs2p-Cdc50p complex (without SEC-HPLC), but we verified that this additional step left these properties essentially unchanged (data not shown).

**Figure 5:**
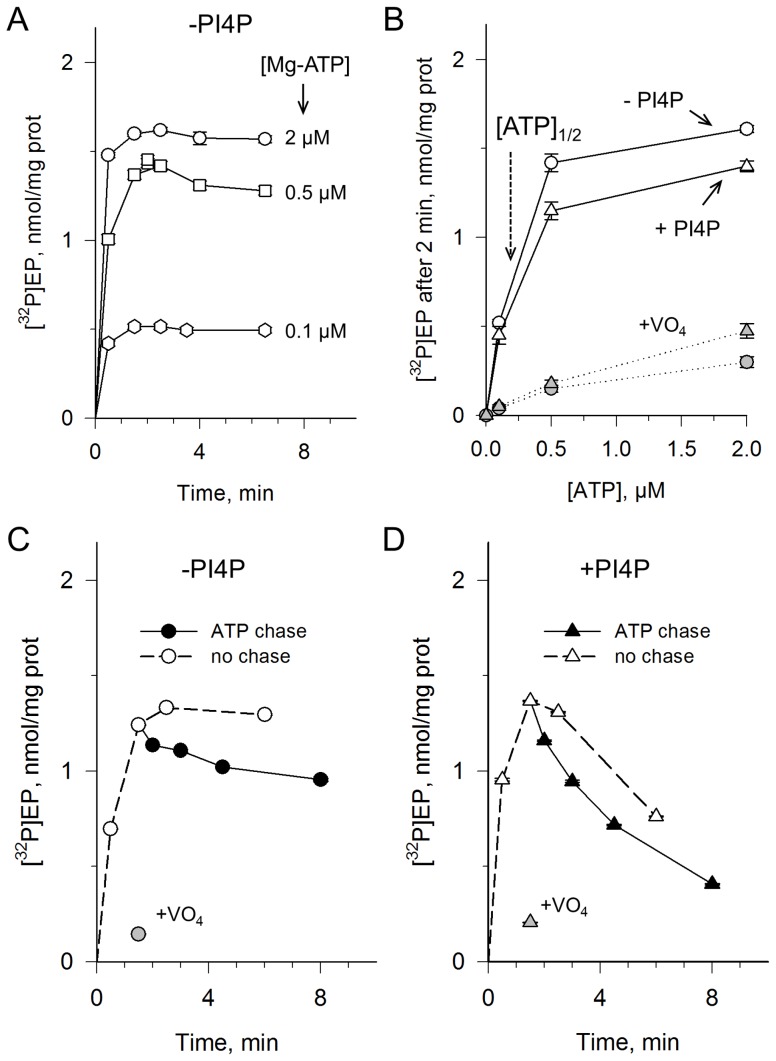
Phosphorylation and dephosphorylation properties of the purified Drs2p-Cdc50p complex. **(A)** Time course of ATP-dependent phosphorylation of the purified Drs2p-Cdc50p complex at various concentrations of [γ-^32^P]ATP. The streptavidin-purified and Ni^2+^-TED-treated sample, diluted to about 50 µg/mL in SSR on ice in the presence of 1 mg/mL DDM and 0.05 mg/mL POPS, was supplemented with [γ-^32^P]ATP (typically 4 µL of concentrated ATP was added to 40 µL enzyme) and brought to 30°C for various periods before acid quenching and filtration. **(B)** ATP-dependence of the maximal phosphorylation level, in the absence (open circles) or presence (open triangles) of PI4P at 0.025 mg/mL. In some cases, the purified sample had been first pre-incubated with 1 mM vanadate (grey symbols). **(C and D)** The streptavidin-purified sample (here before Ni^2+^-TED treatment, and diluted to about 60 µg/mL) was supplemented with 0.5 µM [γ-^32^P]ATP in the absence **(C)** or presence **(D)** of PI4P, and phosphorylation at 30°C was measured (white symbols). Grey symbols refer to experiments performed in the presence of vanadate. To trigger ATP-induced chase of the radioactively-labelled phosphoenzyme, 1 mM non-radioactive Mg-ATP was added after 1.5 minute (black symbols, in the absence of vanadate).

Firstly, the purified Drs2p-Cdc50p complex is efficiently phosphorylated from [γ-^32^P]ATP, up to a forty-fold higher level than solubilized crude membranes (about 1.6 nmol/mg in Figure 5A, compared with 0.04 nmol/mg in Figure 1D). We also found that the observed phosphorylation level was already nearly saturated at a Mg-ATP concentration of 0.5 µM (Figure 5A). This sub-micromolar apparent affinity for Mg-ATP showed up both in the absence and presence of PI4P, whereas in the presence of vanadate the apparent affinity for ATP was much lower (Figure 5B).

Secondly, we found that after a chase with non-radioactive Mg-ATP in the presence of DDM and PS only, slow dephosphorylation of the purified Drs2p took place (Figure 5C). Similar to the situation for Drs2p in crude membranes, this dephosphorylation was accelerated by the additional presence of PI4P, with an estimated decay half-time of about 3–4 minutes (Figure 5D). The purified Drs2p-Cdc50p complex has therefore retained the phosphorylation and dephosphorylation properties previously demonstrated in native or solubilized crude membranes. It is of note that in the presence of PI4P, maximal phosphorylation was soon followed by a decline in the phosphoenzyme level even in the absence of any ATP chase (Figure 5D): the more transient phosphorylation level observed in the presence of PI4P, compared to that in its absence (Figure 5C), is qualitatively consistent with the idea that a faster rate of dephosphorylation in the presence of PI4P results in a faster exhaustion of [γ-^32^P]ATP (here used at a total concentration of only 0.5 µM).

### The WT Drs2p-Cdc50p complex exhibits a PI4P-dependent ATPase activity, while for inactive constructs, only PI4P-independent activity is observed

**Figure 6:**
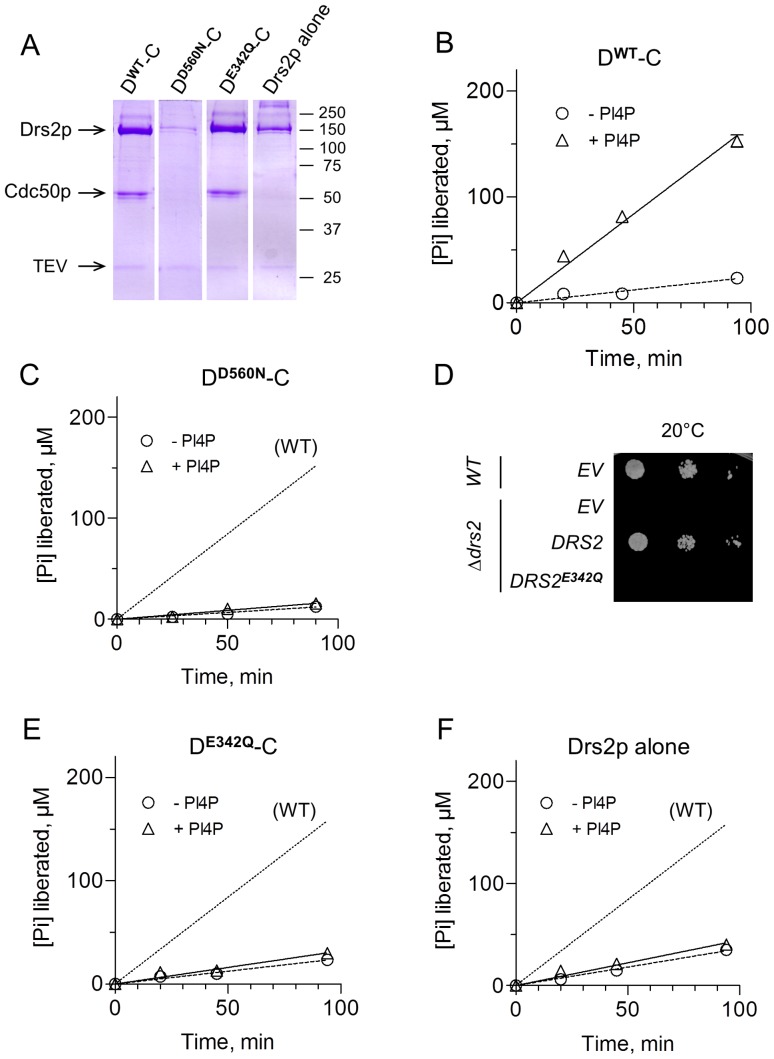
ATPase activities of various streptavidin-purified samples. **(A)** Coomassie Blue staining after SDS-PAGE of purified wild-type complex (D^WT^-C), D560N variant (D^D560N^-C), E342Q variant (D^E342Q^-C), and wild-type Drs2p expressed alone. **(B–C and E–F**) The ATPase activity of the same samples (after 5-fold dilution resulting in about 60 µg/mL Drs2p in the case of the WT enzyme) was measured at 30°C in a KNG medium supplemented with 1 mg/mL DDM, 0.025 mg/mL PS and 1 mM Mg-ATP, in the absence (circles and dashed lines) or presence (triangles and continuous lines) of 0.025 mg/mL PI4P. The dotted line in panels C-F is given for easier comparison with results for WT. **(D)** Functional complementation of the temperature-sensitive phenotype of *Δdrs2* yeast cells. Yeast cells, either wild-type or *Δdrs2*, were transformed with plasmids bearing *DRS2* tagged at its 5′ end, either WT or E342Q. Cells transformed with an empty vector (EV) were used as negative control. Serial dilutions of yeast cells were spotted on plates and incubated at the restrictive temperature of 20°C.

As most of the ATP hydrolyzing contaminants in yeast membranes have presumably been removed during purification, the ATPase activity of the Drs2p-Cdc50p complex could be examined. At 30°C, we were indeed able to detect ATPase activity, and also to reveal, for the WT enzyme, its strong dependence on PI4P (Figure 6B).

We then purified inactive variants, to verify that this PI4P-stimulated ATPase activity was indeed related to Drs2p-Cdc50p. We first generated a Drs2p version mutated on the catalytic aspartate (D560N variant) and co-expressed it together with Cdc50p (D^D560N^-C). We could not detect any PI4P-dependent ATPase activity for the D^D560N^-C complex (Figure 6C), but in this case, Drs2p^D560N^ and Cdc50p had both been expressed and therefore purified at very low levels (ATPase activities were measured at relative protein concentrations identical to those illustrated on the gels in Figure 6A). However, the PI4P-*in*dependent ATPase activity observed for the D560N variant was fairly similar to that of the WT, despite the very large difference in Drs2p concentrations. This suggests that most of this PI4P-independent ATPase activity is not related to the Drs2p-Cdc50p complex.

To rule out the possibility that the PI4P-dependent activity could be mediated by a minor but very active co-purified contaminant, rather than by Drs2p itself, we looked for another mutation that would make Drs2p inactive, without compromising its interaction with Cdc50p. Along those lines, it has been reported that the E342Q mutation of the DGET motif (corresponding to TGES in P2-ATPases), which is known to be critical for dephosphorylation in P-type ATPases (including P4-ATPases, see [25]), preserves the Drs2p-Cdc50p interaction [29]. Upon expression in yeast membranes, the Drs2p^E342Q^ variant could not restore growth of *Δdrs2* cells at low temperatures (Figure 6D), suggesting that the mutation indeed makes Drs2p inactive. After co-expression of such a Drs2p^E342Q^ variant with Cdc50p, the Drs2p^E342Q^-Cdc50p complex could be purified at wild-type levels (Figure 6A). Although its activity in the absence of PI4P was of the same order of magnitude as those observed for the other complexes, no PI4P-dependent activity could be detected for this inactive complex (Figure 6E).

To further strengthen our conclusions, we also purified wild-type Drs2p after its expression in the absence of Cdc50p (Figure 6A). We knew that in the absence of Cdc50p, Drs2p was not able to undergo phosphorylation from [γ-^32^P]ATP [29], [36]. Consistent with expectation, purified Drs2p did not exhibit any detectable PI4P-dependent ATPase activity (Figure 6F), while the ATPase activity measured in the absence of PI4P was similar to that found for WT ATPase. As an additional control, we also purified Cdc50p alone, using a Bad-tagged version of Cdc50p. Again, that purified sample displayed a background PI4P-independent ATPase activity but no detectable PI4P-dependent ATPase activity (data not shown).

From these results we conclude that the PI4P-*in*dependent ATPase activity in the purified samples is most probably due to a contaminant with a substantial specific activity, given that no contaminating band could be visualized on SDS-PAGE (Figure 6A). In contrast, the PI4P-dependent ATPase activity can be unambiguously attributed to the wild-type Drs2p-Cdc50p complex. Consistent with this, it is worth mentioning that after SEC-HPLC, the PI4P-independent activity was reduced compared to the PI4P-dependent activity, consistent with reduction of contaminants during SEC-HPLC (data not shown).

Note that in the experiments displayed in Figure 6B, about 180 µM P_i_ was produced in 100 min for a Drs2p concentration estimated to be 60 µg/mL, implying a specific activity of about 0.03 µmol.min^−1^.mg^−1^ (at 0.025 mg/mL PS and 0.025 mg/mL PI4P). This activity varied somewhat from batch-to-batch, up to 2-fold.

### Characterization of the PI4P-dependent ATPase activity of the purified Drs2p-Cdc50p complex

**Figure 7:**
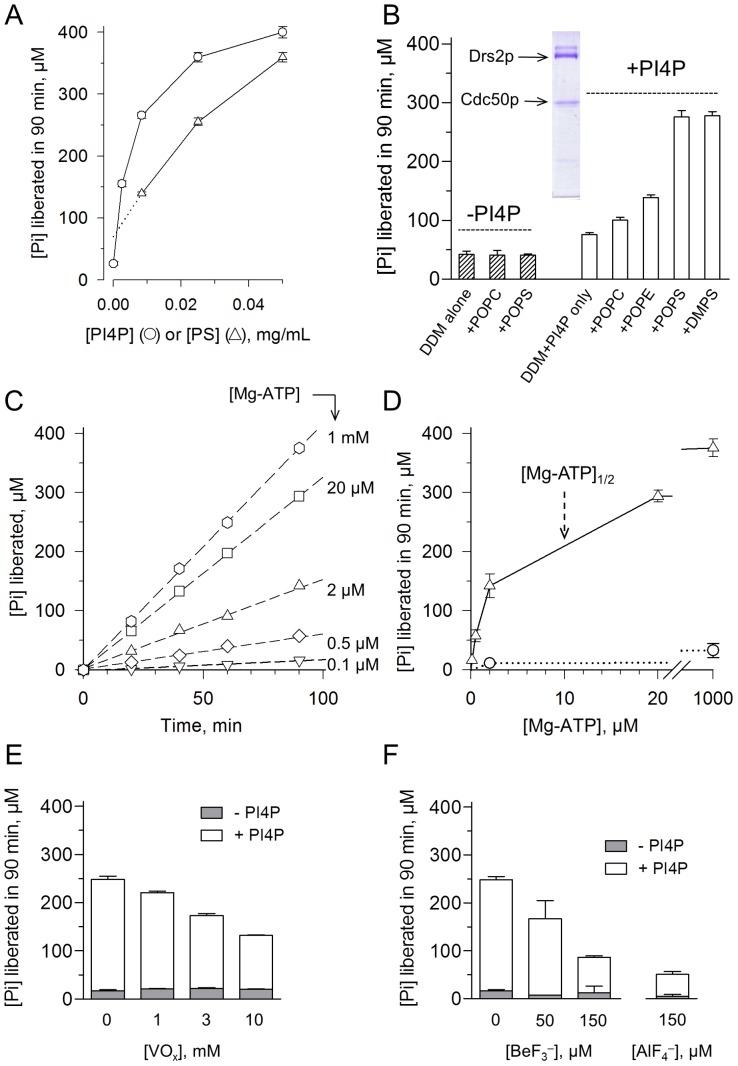
Lipid-dependence and Mg-ATP-dependence of the ATPase activity of the purified sample. **(A)** The ATPase activity of the streptavidin-purified and Ni^2+^-TED-treated sample, diluted to about 50 µg/mL, was measured at 30°C in SSR buffer supplemented with 1 mM Mg-ATP and 1 mg/mL DDM, as well as with PI4P at various concentrations in the additional presence of 0.05 mg/mL POPS (circles), or with POPS at various concentrations in the additional presence of 0.025 mg/mL PI4P (triangles). **(B)** The ATPase activity of a sample purified in the absence of PS (and diluted to about 100 µg/mL) was measured at 30°C in KNG buffer supplemented with 1 mg/mL DDM, 1 mM Mg-ATP, and various lipids (all at 0.05 mg/mL), in the absence (hatched bars) or presence (open bars) of 0.025 mg/mL PI4P. Inset: Coomassie Blue staining of this purified sample after SDS-PAGE. **(C)** The same purified sample as the one for Panel A was used, but in this case the activity was measured at various concentrations of Mg-ATP, in the additional presence of an ATP-regenerating system (and in the presence of 1 mg/mL DDM, 0.05 mg/mL POPS and 0.025 mg/mL PI4P). **(D)** Plot of the results of the experiment displayed in **(C)** for determination of the apparent affinity for ATP, in the presence (open triangles) or absence (open circles) of PI4P. [Mg-ATP]_1/2_ is the concentration of ATP necessary to reach half of the maximal velocity. **(E and F)** After a 1-hour pre-incubation period on ice either with various VO_x_ concentrations (**E**) or with various BeF_3_^−^ or AlF_4_^−^ concentrations (**F**), the ATPase activity of Drs2p-Cdc50p was measured at 30°C in SSR buffer supplemented with 1 mg/mL DDM, 0.05 mg/mL POPS, and 1 mM ATP, in the absence (grey bars) or presence (open bars), of 0.025 mg/mL PI4P.

Now focusing in more detail on the PI4P-dependent ATPase activity of WT Drs2p-Cdc50p complex, this activity appeared to have a number of very attractive features. In the presence of 1 mg/mL DDM and 0.05 mg/mL POPS, it was stimulated by PI4P to a high degree and the apparent [PI4P]_1/2_ was of the order of a few µg/mL (or a few µM) only (Figure 7A) (since PI4P does not distribute equally in the total volume but is mainly found in mixed micelles together with DDM, here used at 1 mg/mL, this apparent affinity is more significantly expressed in terms of PI4P/DDM ratio: [PI4P]_1/2_ was of the order of a few µg of PI4P per mg of DDM). Compared with this high affinity, the apparent affinity for PS (Figure 7A), in the presence of a nearly saturating concentration of PI4P (0.025 mg/mL), was poorer: [PS]_1/2_ was certainly higher than 20 µg/mL. Note that since purification was performed in the presence of PS, the assay medium contained in all cases ∼80 µg/mL of residual PS derived from the purification procedure, hence the uncertainty about the activity in the total absence of PS.

For testing the individual effect of each lipid, we therefore omitted PS during the final steps of the purification procedure (*i.e.* the washing steps which precede TEV addition, as well as the subsequent elution), to better control the amount of PS in the final purified sample. Under the conditions of the present relatively rapid purification protocol, the absence of PS was not deleterious and the enzyme could be purified in a very similar manner (inset to Figure 7B). In the absence of PI4P (Figure 7B), the ATPase activity was independent of the addition of POPC or POPS. In the presence of PI4P (Figure 7B), POPC only had a slightly stimulating effect, POPE had a larger one, and PS had the largest stimulating effect, irrespective of its fatty acyl chain (POPS or DMPS). Addition of PI4P alone to the purified sample had a small effect per se, possibly due to residual PS strongly bound to the purified complex.

Using the enzyme purified in the presence of PS throughout, the apparent affinity for Mg-ATP was about 10 µM in the presence of PI4P (Figure 7C and 7D). This apparent affinity, deduced from overall ATPase measurements, is poorer than the sub-micromolar apparent affinity deduced from steady-state phosphorylation measurements (Figure 4), but for P-type ATPases this is a quite common observation, because of the modulatory effect of ATP on other steps of the functional cycle besides the phosphorylation step itself [51], [52]. Note that an apparent affinity of 10 µM for Mg-ATP for the present purified sample is far stronger than the one previously reported for Drs2p (1.5 mM in [30]).

We also tested the sensitivity to orthovanadate and metallo-fluorides of the PI4P-dependent activity of our purified Drs2p-Cdc50p sample. These classical inhibitors of P-type ATPases did affect the ATPase activity of Drs2p-Cdc50p, but vanadate had a rather poor *K_I_*, in the millimolar range (Figure 7E). In contrast, BeF_3_^−^ and AlF_4_^−^, which are known to be reactive with E2-like states of other P-type ATPases (*e.g.* [35]), significantly inhibited the ATPase activity of Drs2p-Cdc50p for concentrations as low as 150 µM (Figure 7F).

### PI4P protects the purified Drs2p-Cdc50p complex from irreversible denaturation under various conditions

We previously found that during overnight incubation at 4°C of P3 membranes solubilized at a high DDM to protein ratio (10∶1, w:w), a ratio likely to result in delipidation of the solubilized proteins, Drs2p slowly lost its ability to get phosphorylated [36]. We now examined the stability of the streptavidin-purified complex to identify optimal conditions for future studies (*e.g.* for crystallization).

**Figure 8:**
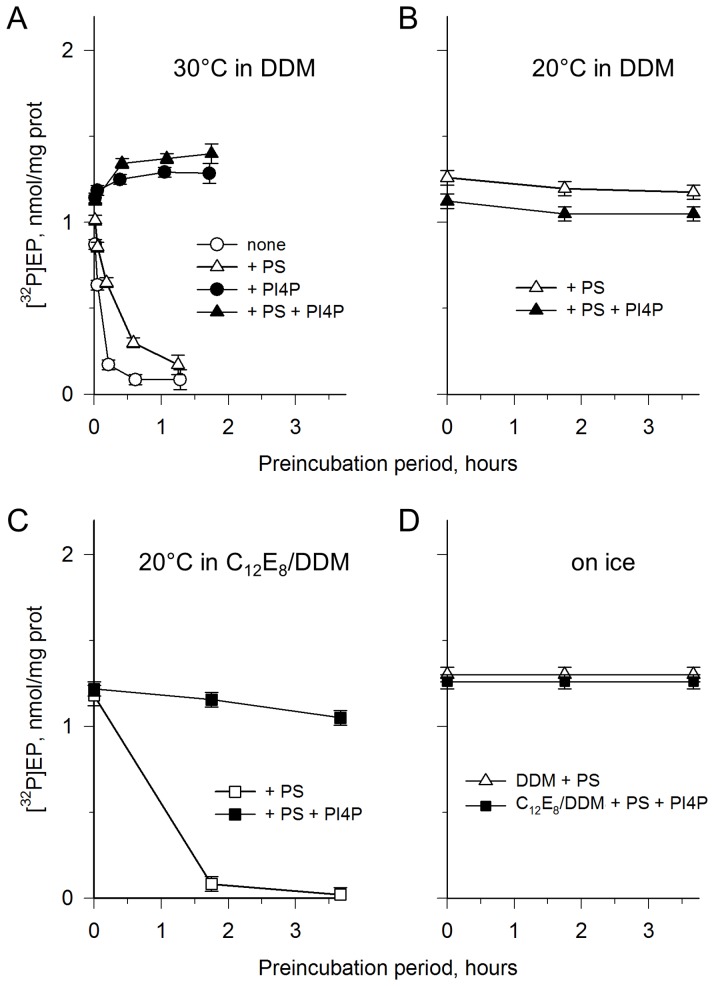
Protection of the purified Drs2p-Cdc50p complex from irreversible denaturation. **(A)** The streptavidin-purified sample (before treatment with Ni^2+^-TED) was diluted to 17.5 µg/mL into KNG buffer at 30°C, supplemented with 1 mg/mL DDM, in the absence (circles) or presence (triangles) of 0.025 mg/mL POPS, and in the absence (open symbols) or presence (closed symbols) of 0.025 mg/mL PI4P. After various periods of incubation, residual phosphorylation was measured 1 min after addition of [γ-^32^P]ATP. **(B–D)** The streptavidin-purified sample (here after treatment with Ni^2+^-TED), was pre-incubated for various periods at about 150 µg/mL in SSR buffer supplemented with various detergents and lipids at different temperatures, before dilution with two volumes of DDM- and lipid-containing SSR buffer, such that in all cases final conditions included 1 mg/mL DDM, 0.05 mg/mL POPS, and 0.025 mg/mL PI4P. 0.5 µM [γ-^32^P]ATP was added and the residual ability of the sample to get phosphorylated was measured (after 1.5–2 min at 30°C). Pre-incubation conditions: **(B)** 20°C, 0.75 mg/mL DDM + 0.025 mg/mL POPS, in the absence (open triangles) or presence (closed triangles) of 0.025 mg/mL PI4P. **(C)** 20°C, 0.75 mg/mL DDM + 0.75 mg/mL C_12_E_8_ + 0.025 mg/mL POPS, in the absence (open squares) or presence (closed squares) of 0.025 mg/mL PI4P. **(D)** On ice, 0.75 mg/mL DDM + 0.75 mg/mL C_12_E_8_ + 0.025 mg/mL POPS in the presence of 0.025 mg/mL PI4P (closed squares), or 0.5 mg/mL DDM + 0.025 mg/mL POPS alone (open triangles).

When incubation in the presence of DDM took place at 30°C, the Drs2p-Cdc50p complex lost its phosphorylation ability very rapidly (over minutes) in the absence of PS (open circles in Figure 8A). The presence of PS exerted significant, but only partial, protection (Figure 8A). DOPC was even less effective than POPS (data not shown). In contrast, we found that the presence of PI4P afforded complete protection at 30°C, and this was in fact true both in the presence and the absence of POPS (Figure 8A).

At 20°C, PS alone was sufficient to make the Drs2p-Cdc50p complex fairly stable over 3 hours in the presence of DDM (Figure 8B), and additional PI4P was therefore not needed. This was however no longer the case in the presence of the more deleterious C_12_E_8_, and in this case the additional presence of PI4P afforded significant stabilization (Figure 8C). Note that in this experiment a significant amount of residual DDM was left together with C_12_E_8_, resulting in a C_12_E_8_/DDM ratio of about 1/1 (w/w).

At 4°C, PI4P allowed Drs2p to remain perfectly stable in the same C_12_E_8_/DDM/PS environment (Figure 8D), just as stable as in the presence of PS alone in DDM (Figure 8D).

Protection of the Drs2p-Cdc50p complex by PI4P, either in the presence of C_12_E_8_ or in DDM at a temperature higher than 4°C, was also checked by measuring the residual ability of the complex to hydrolyze ATP in the presence of PI4P (not only to get phosphorylated from it). The residual ATPase activities were consistent with the residual phosphorylation levels with respect to the ability of PI4P, in addition to POPS, to protect the protein from DDM- or C_12_E_8_-induced damage. In fact, the ATPase activity was even slightly more resistant to inactivation than phosphoenzyme formation (Figure S2). This strong stabilizing effect of PI4P, here demonstrated for both aspects of Drs2p-Cdc50p function, should be of major help for future crystallization of the complex.

## Discussion

In this study, we devised a robust procedure for the purification in high-yield of a yeast lipid flippase, the Drs2p-Cdc50p complex. Because Cdc50 proteins appear to be closely associated with P4-ATPase maturation and function [14], [29], [53], [54], [55], [56], [57], [58], we previously chose to clone *DRS2* and *CDC50* genes into the same expression plasmid. The purification procedure described here represents an additional major step forward for obtaining enough purified material for future structural and functional characterization of the Drs2p-Cdc50p complex.

This procedure is relatively simple and efficient, as most other yeast proteins are eliminated by a single step affinity chromatography (Figure 2), and, most importantly, as it keeps associated (Figure 3) the two co-expressed partners of the complex in what appears to be a 1∶1 ratio (previous reports on the purification of Drs2p or other P4-ATPases have not all been optimal in this regard [29], [30], [57], [59]). The reason why the present protocol yields a purified fraction that mostly contains Drs2p and Cdc50p in complex might be related to the fact that unlike previous attempts, we here chose to clone *DRS2* and *CDC50* genes on a single co-expression plasmid, rather than on two different plasmids, with both genes under the control of the same galactose-inducible promoter. By circumventing a frequent behavior of 2 µ-based plasmids, namely an unequal number of plasmids in each cell, our co-expression system has probably favored a balanced expression of Drs2p and Cdc50p in yeast cells. Our choice of DDM may also have helped keeping the complex intact during the purification procedure.

The purified proteins have the expected sizes, and the very marginal fraction of aggregated protein can be successfully removed by size-exclusion chromatography (Figure 4). The purified complex retains the functional phosphorylation and dephosphorylation properties that can be measured in crude membranes before their solubilization (Figure 5 *vs* Figure 1). Importantly, the ATPase activity of the purified sample is greatly stimulated by the simultaneous presence of PS and the phosphoinositide lipid PI4P (Figure 7), as previously reported [31]. In fact, only the PI4P-dependent ATPase activity can be specifically attributed to active Drs2p-Cdc50p complex, as mutations of either the D560 catalytic aspartate in the phosphorylation motif, or the E342 residue in the DGET motif – whose homologous mutation results in an inactive ATPase in other P-type ATPases [25], [60] – abrogated the PI4P-stimulated activity but left a significant PI4P-independent activity (Figure 6). On the basis of a recent study by Graham's laboratory suggesting that PI4P relieves at least part of the auto-inhibition of Drs2p by its C-terminus, whereas it has a more limited impact on C-terminally truncated forms [31], this would be consistent with our purified fraction mainly containing full-length Drs2p together with Cdc50p. The fraction eluted after affinity chromatography also contains a few contaminant proteins (*e.g.* those contributing to PI4P-independent activity), but they are present in very small quantities (Figure 3A and Figure 6A). These contaminants can be further reduced by SEC-HPLC, with a concomitant reduction of the ratio of PI4P-independent to PI4P-dependent ATPase activities.

The purified Drs2p-Cdc50p complex has a high affinity for its regulator, PI4P (Figure 7). It also retains a high affinity for Mg-ATP, with sub-micromolar apparent affinity with respect to phosphoenzyme formation and micromolar apparent affinity with respect to overall ATPase activity (Figure 5 and Figure 7). These high apparent affinities for PI4P and Mg-ATP speak in favor of the structural integrity of the Drs2p-Cdc50p complex. The poorer apparent affinity for ATP previously described for purified Drs2p (about 1.5 mM, see [30]) might be related to the use in that case of C_12_E_9_ for solubilization, as in our hands the related C_12_E_8_ detergent proved rather inactivating (Figure S2 and [36]). Alternatively, the discrepancy might be due to the presence of some Cdc50p-unbound Drs2p in that study [30]. The poor apparent potency of vanadate which we measured for inhibition of the PI4P-dependent ATPase activity (Figure 7E) could at first seem puzzling, but it might simply be taken as an indication that during turnover in the presence of a large ATP concentration (1 mM for ATPase activity measurements), most of the Drs2p-Cdc50p complex resides in a state which is not much sensitive to vanadate. A similar situation has been described for well-known P-type ATPases (*e.g.* SERCA1a), because vanadate is thought to recognize efficiently only certain conformations of the pump during its turnover [45], [61], [62]. Furthermore, our purified sample displayed an apparent affinity for vanadate in the micromolar range with respect to phosphoenzyme formation (data not shown), which is a value similar to that previously described for Drs2p embedded in yeast membranes [36].

At this point, it is useful to discuss another remarkable result of our experiments, namely the fact that the ATPase activity we measured for purified Drs2p-Cdc50p was rather slow, typically around 30–60 nmol.mg^−1^.min^−1^ at 30°C in the presence of 1 mM Mg-ATP, 1 mg/mL DDM, 0.025 mg/mL POPS and 0.025 mg/mL PI4P, and in fact slower than previously reported [30], [31]. This slow rate goes along with the previously noted very slow Drs2p dephosphorylation measured after an ATP chase, including in crude membranes ([36] and this study). Compared with ATP hydrolysis and transport rates measured for well-known P2-type transport ATPases, *e.g.* the Na^+^,K^+^-ATPase or SERCA1a under similar conditions, these are very slow rates. For example, under similar conditions (except for the absence of PI4P), we measured an activity for SERCA1a at least ten-fold higher than that of Drs2p-Cdc50p (data not shown). Another P4-type ATPase, the bovine ATP8A2, was recently described to hydrolyze ATP at rates close to 150 µmol.mg^−1^.min^−1^ at 37°C and 7.5 mM Mg-ATP [26].

This raises a number of issues. If the true activity of our purified Drs2p-Cdc50p complex is slow, could it be that it lacks a critical P4-ATPase co-factor? For instance, whether Gea2p, which has been shown to associate with Drs2p [15] and to play a role in the Drs2p flippase machinery [16] co-purifies with the Drs2p-Cdc50p complex may be worth investigating, although we do not expect this to be a critical component as no significant stimulation of the Drs2p activity by the Gea2p Sec7 domain was observed [31]. We think that it is reasonable to anticipate that the Drs2p-Cdc50p complex indeed has a fairly slow turnover rate (as suggested by its rates of dephosphorylation and ATP hydrolysis measured here), because in yeast *trans*-Golgi or secretory vesicle membranes, PS seems to be transported quite slowly, over hours [63], [64], *i.e.* at a rate probably consistent with a slow rate of ATP hydrolysis, at least assuming a coupling ratio of ∼1 between ATP hydrolysis and lipid transport. A slow transport with a fast ATPase activity would in fact imply poor coupling.

Conversely, the reason why other P4-type flippases, like bovine ATP8A2 found for example in membranes of retinal photoreceptors, hydrolyze ATP and get dephosphorylated during turnover extremely rapidly (the reported hydrolysis rate of 150 µmol.mg^−1^.min^−1^ [25], [26] implies a turnover number around 350 s^−1^, i.e. that lipid transport occurs within milliseconds) is unclear. It was previously suggested [36], [65] that perhaps rapid active transport of lipid was needed in specific types of membranes (like the one from which ATP8A2 originates) to efficiently counterbalance rapid dissipation of lipid asymmetry by ATP-independent flippases located in those membranes (*e.g.* opsin in retinal photoreceptors [65]). However, flipping of lipids by opsin is thought to occur at a rate faster than 10,000 per second [65], [66], making it rather unlikely that ATP8A2 can efficiently counteract randomization of lipids by opsin. Therefore, the question remains open.

From a mechanistic or molecular point of view, it might be speculated that the slow activity of Drs2p could be due to auto-inhibition by its C-terminal and/or N-terminal extensions (as often observed in other P-type ATPases). The existence of such auto-inhibition of Drs2p by its C-terminal end has been suggested recently [31]. Auto-inhibition by C- or N-terminal extensions might even be a conceivable explanation for the fact that our purified Drs2p enzyme is significantly slower than Drs2p purified in other labs. Both in our hands (Figure 1A) and in the Graham lab, for instance, various types of truncations of the overexpressed Drs2p have been detected. Assuming that some very limited (and thus undetectable) truncation occurs, to a variable extent, and that this truncation partly relieves the auto-inhibition of Drs2p, this might significantly influence the overall ATPase activity of the purified Drs2p-Cdc50p complexes, both in our lab (where we found up to 2-fold variability in ATP hydrolysis rates) and in other labs. Future studies aiming at deciphering the Drs2p-Cdc50p transport mechanism should help to resolve this apparent discrepancy.

Finally, this work also presents a finding which might be of major significance. A critical parameter for successful crystallogenesis of a membrane protein is the choice of the detergent to be used, because among other effects it influences the stability of the protein [67] and for several P-type ATPases, C_12_E_8_ has proven superior to other detergents in terms of crystal growth [20], [21], [22]. Therefore, our finding that phosphoenzyme formation from ATP and the overall ATPase activity of Drs2p-Cdc50p are strongly stabilized by PI4P in the presence of both DDM or C_12_E_8_ (Figure 8) is an important step towards obtaining well-diffracting, functionally-relevant crystals of a particular and presumably important conformation of the Drs2p-Cdc50p complex.

## Supporting Information

Figure S1 **The vast majority of Drs2p is in complex with Cdc50p, as analyzed by western-blotting.** The same samples as those in Figure 3 were loaded onto a 10% SDS-PAGE and the gel was probed with a α-Drs2p antibody or a Histidine probe. For D-_His10_C, the E_s_ sample was further diluted 3-fold and 10-fold (E_s_/10 and E_s_/3) and aliquots were loaded for comparison with the FT sample.
(TIF)

Figure S2 **Protection by PI4P of the ATP hydrolysis ability of the purified Drs2p-Cdc50p complex.** The streptavidin-purified sample (after treatment with Ni^2+^-TED), was pre-incubated for various periods at ∼150 µg/mL in SSR buffer supplemented with various detergents and lipids and at different temperatures. These samples were then diluted with two volumes of DDM- and lipid-containing SSR buffer, such that in all cases final conditions included 1 mg/mL DDM, 0.05 mg/mL POPS, and 0.025 mg/mL PI4P. The ATPase activity was then measured at 30°C, in the presence of 1 mM Mg-ATP. Pre-incubation conditions: 20°C, 0.75 mg/mL DDM + 0.025 mg/mL POPS, in the absence (open triangles) or presence (closed triangles) of 0.025 mg/mL PI4P; 20°C, 0.75 mg/mL DDM + 0.75 mg/mL C_12_E_8_ + 0.025 mg/mL POPS, in the absence (open squares) or presence (closed squares) of 0.025 mg/mL PI4P.
(TIF)
