# Supplementary figures and images for: A High-Yield Co-Expression System for the Purification of an Intact Drs2p-Cdc50p Lipid Flippase Complex, Critically Dependent on and Stabilized by Phosphatidylinositol-4-Phosphate
Source: PLoS One. 2014 Nov 13;9(11):e112176. doi: 10.1371/journal.pone.0112176 (PMC4230938; doi:10.1371/journal.pone.0112176)

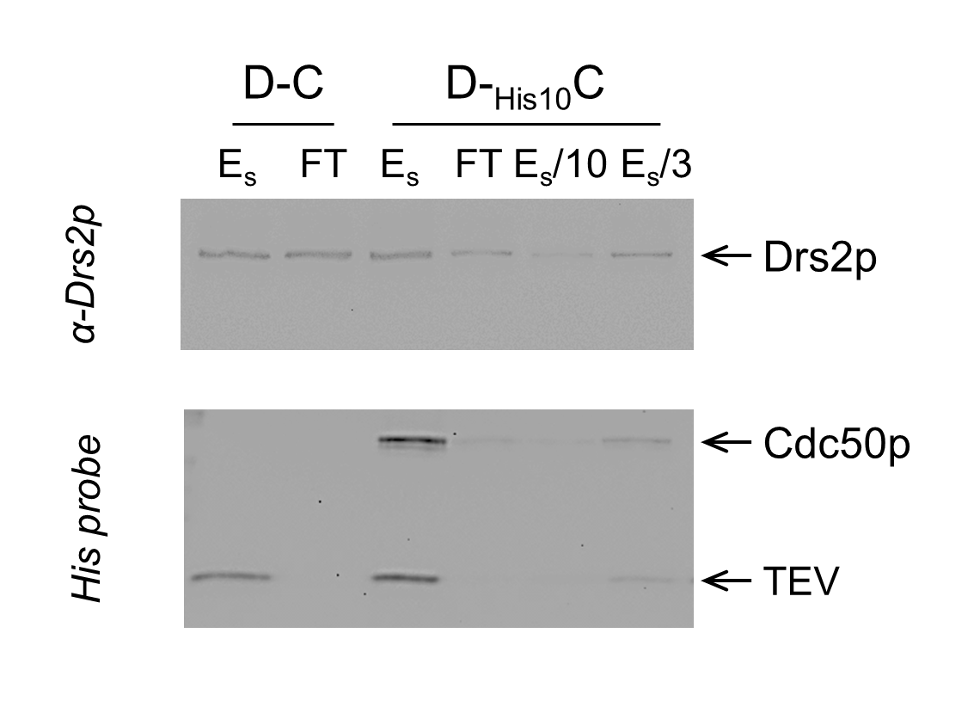

Supplement: Figure S1 — The vast majority of Drs2p is in complex with Cdc50p, as analyzed by western-blotting. The same samples as those in Figure 3 were loaded onto a 10% SDS-PAGE and the gel was probed with a α-Drs2p antibody or a Histidine probe. For D-His10C, the Es sample was further diluted 3-fold and 10-fold (Es/10 and Es/3) and aliquots were loaded for comparison with the FT sample. (TIF) [file pone.0112176.s001.tif]

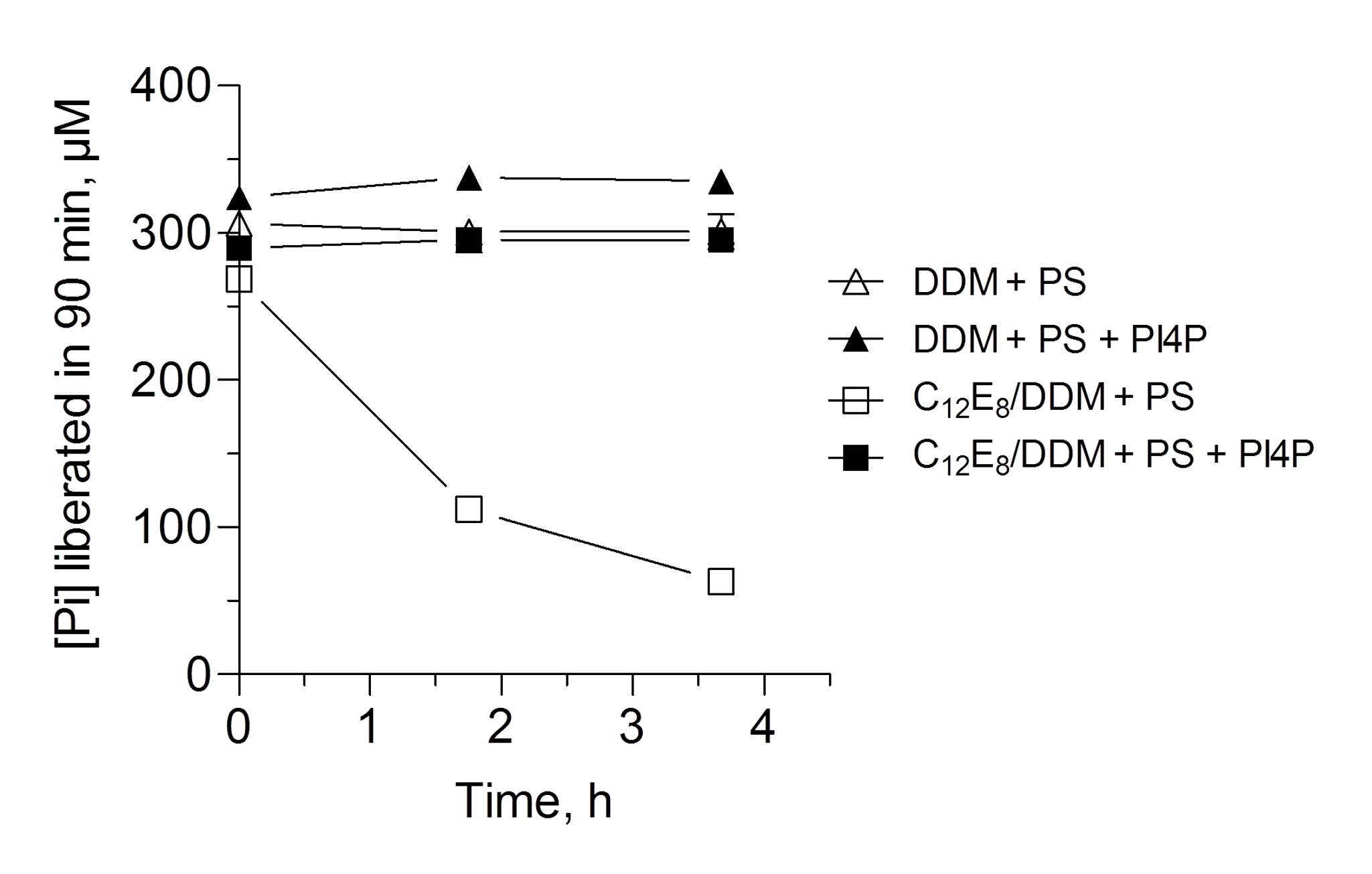

Supplement: Figure S2 — Protection by PI4P of the ATP hydrolysis ability of the purified Drs2p-Cdc50p complex. The streptavidin-purified sample (after treatment with Ni2+-TED), was pre-incubated for various periods at ∼150 µg/mL in SSR buffer supplemented with various detergents and lipids and at different temperatures. These samples were then diluted with two volumes of DDM- and lipid-containing SSR buffer, such that in all cases final conditions included 1 mg/mL DDM, 0.05 mg/mL POPS, and 0.025 mg/mL PI4P. The ATPase activity was then measured at 30°C, in the presence of 1 mM Mg-ATP. Pre-incubation conditions: 20°C, 0.75 mg/mL DDM + 0.025 mg/mL POPS, in the absence (open triangles) or presence (closed triangles) of 0.025 mg/mL PI4P; 20°C, 0.75 mg/mL DDM + 0.75 mg/mL C12E8 + 0.025 mg/mL POPS, in the absence (open squares) or presence (closed squares) of 0.025 mg/mL PI4P. (TIF) [file pone.0112176.s002.tif]
